# Two host‐plant strains in the fall armyworm

**DOI:** 10.1111/1744-7917.13346

**Published:** 2024-03-04

**Authors:** Kiwoong Nam, Nicolas Nègre, Clara Ines Saldamando Benjumea

**Affiliations:** ^1^ DGIMI, Université de Montpellier, INRAE Montpellier France; ^2^ Grupo de Biotecnología‐Vegetal UNALMED‐CIB, Laboratorio de Ecología y Evolución de Insectos 16–223 Facultad de Ciencias, Departamento de Biociencias, Universidad Nacional de Colombia Medellín Colombia

**Keywords:** fall armyworm, host‐plant adaptation, incipient speciation, *Spodoptera frugiperda*

## Abstract

The fall armyworm (*Spodoptera frugiperda*) is one of the major pest insects damaging diverse crops including cotton, corn, rice, and sorghum. Fall armyworms have been identified as two morphologically indistinguishable strains, the corn strain, and the rice strain, named after their preferred host‐plants. Although initially recognized as host‐plant strains, there has been an ongoing debate regarding whether the corn and rice strains should be considered as such. In this article, we present arguments based on recent population genomics studies supporting that these two strains should be considered to be host‐plant strains. Furthermore, host‐plant adaptation appears to be a driving evolutionary force responsible for incipient speciation in the fall armyworm.

## Graphical abstract

The existence of two sympatric strains within the fall armyworm, a lepidopteran species, has been well‐documented for nearly four decades. However, the underlying evolutionary mechanism responsible for the genetic differentiation between these strains has remained a subject of intense debate. This review highlights recent evolutionary genomic studies, demonstrating the role of host‐plant adaptation as the driving force behind the incipient speciation between these strains.

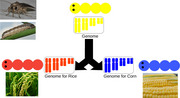

## Introduction

The fall armyworm (FAW, *Spodoptera frugiperda*, Lepidoptera: Noctuidae) is one of the major pest insects to diverse crops, including maize, sorghum, cotton, sugar cane, and rice due to its high polyphagy (Montezano *et al.*, 2018). Originally native to North and South America, the invasion of FAW was first documented in West Africa in 2016 (Goergen *et al.*, 2016). Since then, FAW has rapidly spread to numerous regions in the Old World, including Sub‐Saharan Africa, Middle East Asia, South Asia, Southeast Asia, East Asia, Oceania, Egypt, Cyprus, the Canary Islands (Rwomushana, 2019), and recently to Turkey and Greece (https://gd.eppo.int/taxon/LAPHFR). Invasive populations of FAW caused severe losses in corn production, particularly in Sub‐Saharan Africa, where FAW is responsible for approximately 21%–53% of corn yield losses (Day *et al.*, 2017). This is of great concern, as corn provides at least 30% of the caloric intake in the region (Nuss & Tanumihardjo, 2010). The management of FAW typically involves the use of synthetic insecticides or *Bt* (*Bacillus thuringiensis*) toxins via genetically modified crops or direct spraying. However, field‐evolved resistance to synthetic insecticides (Bai‐Zhong *et al.*, 2020; Gui *et al.*, 2020; Zhao *et al.*, 2020; Guan *et al.*, 2021; Lv *et al.*, 2021; Zhang *et al.*, 2021) or *Bt* (Blanco *et al.*, 2010; Storer *et al.*, 2010; Chandrasena *et al.*, 2018) has often been observed, challenging the management of FAW.

## Two strains in the fall armyworm

FAW is composed of two strains, corn strain (sfC) and rice strain (sfR) with differentiated ranges of host‐plants (Pashley, 1986; Pashley & Martin, 1987). As the names indicate, sfC prefers corn, sorghum, and cotton, while sfR prefers rice, grasses, and millet. sfC and sfR are found sympatrically in native populations while invasive populations were reported to be sfC (Yainna *et al.*, 2022) (please see below for relevant debate). Reciprocal transplant experiments showed that sfC and sfR strains exhibit different levels of fitness on different host‐plants, suggesting differentiated adaptation to host‐plants (Orsucci *et al.*, 2022). Furthermore, sfC and sfR genomes have different sets of host‐plant genes, involving the detoxification of plant toxins (or insecticides) and the chemosensation or digestion of plant molecules (Gouin *et al.*, 2017). Since these two strains are morphologically indistinguishable, molecular markers have been used for their identification. These markers include the Z chromosome triosephosphate isomerase (TPI) gene (Nagoshi, 2010), the mitochondrial cytochrome c oxidase subunit 1 (mt‐COX1) gene (Pashley, 1989), and the sex chromosome FR repeat (Lu *et al.*, 1994).

Even though sfC and sfR were first presented as *host‐plant strains* in the 1980s (Pashley, 1988), it remains unclear whether these two taxonomic identities refer to host‐plant strains. sfC and sfR have been reported to have allochronic mating time (Pashley *et al.*, 1992; Schöfl *et al.*, 2011, 2009; Saldamando‐Benjumea *et al.*, 2014; Hänniger *et al.*, 2017; Tessnow *et al.*, 2022), and Tessnow *et al.* (2022) even suggested that the corn and rice stains should be considered to be *allochronic strains* rather than host‐plant strains. Furthermore, several studies focus solely on the molecular markers of strains without considering the host‐plants. For example, if invasive FAWs have sfR‐type mt‐COX1 genes, these FAWs were reported to be sfR even though these samples were collected from corns (Herlinda *et al.*, 2022; Yousaf *et al.*, 2022). In this case, sfC and sfR are *genetic strains*, without explicit association with phenotypes. Imperfect correlation between marker sequences and host‐plants often have been interpreted to be the infidelity of host‐plants (Juárez *et al.*, 2012), rather than the reliability of marker sequences, posing a skepticism that sfC and sfR are indeed host‐plant strains. In short, it remains unclear whether sfC and sfR should be considered to be host‐plant strains, as initially suggested, or something else (Nagoshi & Meagher, 2022).

## Host‐plant strains

Assuming that the term “strain” can be interchanged with “race” to denote a biotype generated along the speciation continuum, we will use the definition of host‐plant strains according to the one by Drès and Mallet (2002), such that “host races are genetically differentiated, sympatric populations of parasites that use different hosts, and between which there is appreciable gene flow.” Feder *et al.* also aptly noted that genetic differentiation should manifest as discernible “*genotype clusters*,” characterized by a bimodal differentiation pattern across multiple loci within a genome (Feder *et al.*, 2012a). Therefore, according to this definition, sfC and sfR should be considered to be host‐plant strains if genetic differentiation is observed from multiple loci in a genome between sympatric sfC and sfR populations utilizing different host‐plants even in the presence of gene flow. Population genomics analyses can be useful tools to test genetic differentiation at multiple loci with gene flow through whole genome analyses.

Moreover, population genomics approaches have the capacity to unveil subtle genetic variations stemming from slight differences in fitness, which might not be detectable through laboratory observations. Disparities in host‐plant fidelity have often not been readily apparent under laboratory conditions between sfC and sfR strains, which have raised skepticism about whether sfC and sfR are indeed host‐plant strains (Nagoshi & Meagher, 2022). However, this skepticism might be attributed solely to insufficient statistical power for detecting potentially existing subtle differences with a limited sample size in a laboratory.

**Fig. 1:**
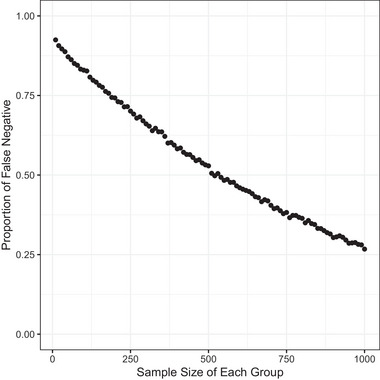
Testing the power to detect small differences in fitness. We considered two groups in which a quantitative trait related to fitness differs by only 1%. The trait follows a normal distribution, and the standard deviation is 10% of the mean. With varying sample sizes for each group (*x*‐axis), we tested whether a one‐tailed *t*‐test can detect the existing differences with a 5% significance level with 10,000 replications. We then calculated the proportion of false positives among the replications (*y*‐axis).

To illustrate, we conducted a simple simulation involving two groups characterized by quantitatively different traits, often employed to estimate fitness (e.g., generation time or body weight). These traits follow normal distributions, with the mean differing by only 1% between the two groups. The standard deviation is set at 10% of the mean value. Subsequently, we assessed whether a one‐tailed *t*‐test could detect differences in the traits with *P*‐values lower than 0.05, involving varying sample sizes in 10,000 replications. We then calculated the probability of false negatives. Fig. 1 shows that, even when the sample size is 1,000 for each group, the probability of false negatives was 26.73%. When the sample size was 100 for each group, this probability was as high as 82.96%. Given that laboratory experiments with more than one thousand individuals are impractical, detecting a very slight fitness difference in such experiments is very challenging.

In natural populations, however, a very subtle difference in fitness may generate a clear pattern of genetic differentiation. To illustrate, let's consider a scenario where a population experienced positive selection leading to the fixation of a beneficial mutation with a selective coefficient of merely 1%. Utilizing the formula (29) as presented by Smith and Haigh (1974), the associated selective sweep will decrease genetic diversity over a genetic distance of 200 × 0.01/[3 × ln (10^6^)] cM = 0.048 cM, with the assumption that the population size is 10^6^. Assuming a recombination rate of 2.97 cM/Mb (Yamamoto *et al.*, 2008), this genetic distance translates to 0.048/2.97 Mb = 16.16 Kb. Thus, each instance of positive selection involving a very slightly advantageous mutation will leave discernible footprints of selective sweep across 16.16 Kb of neutral sequences, in addition to substitution of the targeted nucleotide position. Therefore, population genomics approaches can be useful to test the existence of a diverging evolutionary force in FAW, particularly when the effect could be undetectable in a laboratory with a limited number of investigated samples.

Fiteni *et al.* (2022) conducted a population genomics analysis using larvae collected from corn (sfC‐preferred host‐plants) and grasses (sfR‐preferred host‐plants) in the United States. They demonstrated that almost the whole genome sequences exhibit differentiation between samples collected from different host‐plants. Since this genomic differentiation dominates the population structure of FAW unlike geographic effects, they concluded that sfC and sfR are indeed host‐plant strains. Additionally, they suggested that autosomal divergent selection initiated genetic differentiation between strains, and subsequent divergent selection on the Z chromosome further increased the level of differentiation. Interestingly, they also observed a noticeable hybrid between sfC and sfR, implying the existence of gene flow. These empirical results further suggest that sfC and sfR are indeed host‐plant strains. Moreover, the observation that sfC and sfR exhibit whole genome differentiation implies that the selection pressure exerted by different host‐plants has been substantial enough to trigger significant genetic differentiation across whole genome sequences, through very strong divergent selection targeting a single locus (Barton, 1979; Flaxman *et al.*, 2014) or through the combined effect of mild divergent selection targeting many loci (Barton, 1983; Feder & Nosil, 2010), or the combination of both strong and mild divergent selection.

## Prezygotic reproductive isolations

Several studies on reproductive isolation have been conducted with sfC and sfR, suggesting the evolution of prezygotic isolation through assortative mating. The allochronic mating pattern has been repeatedly reported from the observation that sfC mates early at night and sfR late at night from natural populations from the United States (Pashley *et al.*, 1992; Schöfl *et al.*, 2011, 2009) or from a laboratory colony (Hänniger *et al.*, 2017), while such an allochronic pattern was not detected from populations from Colombia (Saldamando‐Benjumea *et al.*, 2014). Even if sfC and sfR indeed have allochronic mating patterns, these two strains should not be considered allochronic strains because it is still unclear to which extent allochronic mating patterns contributed to genomic differentiation. The association between mating times and strains has been shown only from one or two marker sequences, rather than “*genotype clusters*” (Feder *et al.*, 2012a). Therefore, allochronic strains do not fit the definition of strains.

It should be noted that allochronic mating and differential host‐plant adaptation might not have synergistic or additive effects on increasing the level of genomic differentiation into two groups without the concordant direction of differentiation between assortative mating by allochronic mating and ecological divergent selection through host‐plant adaptation. Recombination between loci determining assortative mating and ecological divergent selection could generate all possible allelic combinations of these loci. In such cases, the direction of differentiation is determined by the relative strength between assortative mating and ecological divergent selection. In this case, divergent selection on these two loci might interfere with each other (Felsenstein, 1981). On the other hand, if a single locus controls both mating time and host‐plant adaptation, then differentiation into two groups may occur readily. Further studies are necessary to assess the contribution of mating time to genomic differentiation in FAW. Until then, sfC and sfR should be considered host‐plant strains.

Two strains also have different proportions of main compounds in sexual pheromones (Groot *et al.*, 2008; Lima & McNeil, 2009; Unbehend *et al.*, 2013, 2014). Volatile components emitted by females are also different as sfC and sfR produce eight and eleven strain‐specific components, respectively, while only nine components are common between strains (Cañas‐Hoyos *et al.*, 2017). However, sfC and sfR should not be considered allopheromonic strains since the existence of differentiated “genotype clusters” based on sexual pheromones has not been demonstrated.

## Postzygotic reproductive isolations

Artificial crosses between female sfC and male sfR individuals revealed severe reductions in fertility (Pashley & Martin, 1987; Velásquez‐Vélez *et al.*, 2011; Dumas *et al.*, 2015), which indicates the presence of intrinsic reproductive isolation. Moreover, hybrids displayed a reduction in fitness, through reductions in egg mass count, larval count, female count, duration of pupal development, adult lifespan, and pupal weight (Velásquez‐Vélez *et al.*, 2011), again implying the existence of intrinsic reproductive isolation. Further studies are needed to understand the contribution of intrinsic reproductive isolation to incipient speciation and the interaction of intrinsic reproductive isolation with host‐plant adaptation.

## Invasive populations

Invasive populations of FAW have been proposed to be hybrids between sfC and sfR, primarily based on the presence of corn sfR‐type at mt‐COX1 and sfC‐type at the Z chromosome TPI in the majority of invasive FAW samples (Nagoshi *et al.*, 2019, 2017, 2022). However, it has been puzzling how these hybrids are predominantly found in crops preferred by sfC in invaded areas. For example, Nagoshi *et al.* (2021) demonstrated through systematic field surveys that FAWs in Ghana and Togo are almost absent from rice or pastures, which are preferred host‐plants of sfR. In East and Southeast Asia, where rice is very widely cultivated and extensive monitoring of rice‐attacking pest insects has been regularly performed (Kim *et al.*, 2018), FAWs are only sporadically detected from rice (for example, Navasero *et al.*, 2023) without notable infestation. Yainna *et al.* (2022) conducted a population genomics analysis using 177 globally sampled individuals to investigate the invasive history of FAW. They observed clear genomic differentiation between sfC and sfR in native areas from the result of principal component analysis. They also showed that invasive and native sfC samples are undifferentiated along the first principal component while these two groups exhibited whole genome differentiation along the second principal component. Based on these findings, they concluded that invasive FAWs should be regarded as sfC strains rather than hybrids. This conclusion explains the observed distribution of host‐plants in invaded areas. Furthermore, their results suggest that invasive FAWs pose a low risk of infesting rice. It is important to note that their results represent an average pattern across the entire genome, and it is still possible that a minority of genomic loci in invasive populations may have been derived from sfR through genetic introgression. In such a case, invasive FAWs could be considered partial hybrids.

## Differentiation within corn strain

As mentioned before, Fiteni *et al.* (2022) observed a distinct pattern of whole genome differentiation between samples from corn (sfC) and pastures (sfR). Interestingly, sfC comprises samples with sfC‐mt‐COX1 and sfR‐mt‐COX1 sequences, referred to as mtA and mtB, respectively, while all these samples exhibit sfC‐TPI marker sequences. The level of genetic differentiation between mtA and mtB was very low (*F*_ST_ = 0.0105), and no random grouping in 100 replications produced a higher *F*_ST_ than 0.0105, indicating statistical significance of genetic differentiation with *P*‐value lower than 0.01. The observed nuclear differentiation between mtA and mtB could be a consequence of potential hybridization between sfC and sfR, leading to the generation of mtB, which exhibits a discordant strain identity between nuclear and mitochondrial markers.

However, principal component analysis showed that mtB samples are not genetically closer to sfR than mtA. Furthermore, the ancestry coefficient analysis demonstrated that mtB does not have a higher proportion of genomic sequences originating from sfR than mtA. Fiteni *et al.* showed that 92.7% of 500 kb windows have *F*_ST_ higher than 0 between mtA and mtB, meaning that the genetic differentiation is not confined to a small number of loci in the genome. Instead, this result indicates that genetic differentiation is observed across almost the entire genome. Nam *et al.* (2020) suggested that the whole genome differentiation between mtA and mtB is the consequence of the genome hitchhiking effect (Feder *et al.*, 2012b), a reduction in effective gene flow across the entire genome due to divergent selection targeting a large number of loci. Please note that sfC and sfR mentioned in Nam *et al.* correspond to mtA and mtB according to the classification of Fiteni *et al.* Taken together, all these results suggest that nuclear differentiation between mtA and mtB is not likely to be the consequence of hybridization events. Instead, Fiteni *et al.* proposed an evolutionary scenario in which the ancestral FAW split into two strains with different host‐plants (sfC and sfR), and sfC further split into two substrains, mtA and mtB.

## Markers

As mentioned above, it is reasonable to use molecular markers to identify strains because sfC and sfR are morphologically indistinguishable (but see Cañas‐Hoyos *et al.*, 2014 and Nagoshi *et al.*, 2020). As stated earlier, Fiteni *et al.* (2022) showed that TPI sequences exhibit a perfect correlation with host‐plants, indicating that the TPI gene can be used to identify host‐plant strains. The observation that samples from cornfields have sfR‐mtCOX1 with sfC‐TPI implies the existence of mtB, rather than the past event of hybridization between sfC and sfR (Prowell *et al.*, 2004). Thus, mitochondrial markers should be used to identify mtA or mtB substrains, rather than sfC or sfR strains.

It should be noted that, even though the correlation was perfect in this study, the original paper on the TPI marker mentions that the sequence of diagnostic positions in TPI genes has imperfect and variable correlations with host‐plants (Nagoshi, 2010). We argue that it is urgently necessary to develop improved marker sequences for the identification of FAW strains through the evaluation of the TPI gene and other candidate genes, especially on the Z chromosomes (Prowell *et al.*, 2004).

## Mitochondrial evolution

The existence of two substrains within sfC seems to contradict the findings of phylogenetic studies based on mt‐COX1 genes. A molecular clock study indicated that the mitochondrial genomes of mtA and mtB diverged approximately 3 million years ago (Kergoat *et al.*, 2012). However, this divergence time does not easily explain the very low level of nuclear genomic differentiation observed between mtA and mtB substrains (Nam *et al.*, 2020; Fiteni *et al.*, 2022). The sequence difference of mitochondrial COX1 genes between mtA and mtB was estimated to be 2.09% (Kergoat *et al.*, 2012), which is nearly the level of inter‐species difference in insects (Hendrich *et al.*, 2010). Therefore, based solely on mitochondrial genomes, mtA and mtB could be considered even different species (Dumas *et al.*, 2015). However, the low level of nuclear differentiation between mtA (or sfC) and mtB (or sfR) makes it unreasonable to consider that these two taxa have distinct gene pools (*F*_ST_ = 0.0105) (Fiteni *et al.*, 2022), which are required for the concept of biological species.

**Fig. 2:**
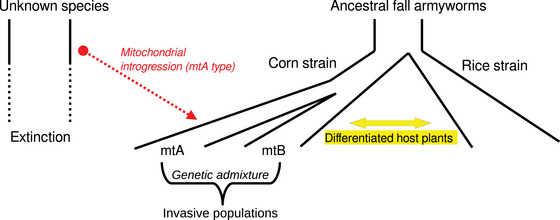
Suggested evolutionary scenario of the FAW. The ancestral fall armyworms with mtB‐type mitochondrial sequences underwent a divergence that led to the generation of corn and rice strains with differentiated host‐plants. Subsequently, the corn strain underwent additional nuclear differentiation, resulting in the generation of two substrains. One of these substrains experienced mitochondrial introgression from an extinct species with mtA‐type mitochondrial sequences. As a result, within the corn strain, two substrains had highly differentiated mitochondrial genomic sequences (mtA and mtB). Invasive populations originated from corn strains with the involvement of genetic admixture between mtA and mtB (Yainna *et al.*, 2022).

A possible explanation for this puzzle is the occurrence of mitochondrial leakage from an extinct species with mtA‐type sequences (Fig. 2). According to this explanation, a sister species of FAW had a mtA‐type sequence in the mitochondria, while the ancestral FAW population had mtB‐type (or sfR‐type) sequences. At some point, genetic introgression from this sister species led to the leakage of mtA‐type mitochondrial sequences into a subgroup of sfC, which subsequently evolved into the extant mtA substrain. This evolutionary scenario could explain the high level of mitochondrial genome differentiation between mtA and mtB within extant sfC individuals. However, two questions remain unanswered in this scenario. First, this scenario does not explain why mitochondrial genomes have no detectable differentiation between mtB and sfR while only nuclear genomes are highly differentiated (Fiteni *et al.*, 2022). Second, the biological significance of the differences between mtA and mtB remains unknown. Orsucci *et al.* (2022) proposed that the host utilization is constrained by different metabolic activities between sfC and sfR through differential mitochondrial gene expressions, and it will be interesting to test the difference in metabolic activities between mtA and mtB in a future study. Furthermore, incompatibilities between the mitochondrial genomes and loci on the sex chromosome might also contribute to the reduction of gene flow in hybrids (Hill, 2015). Addressing these questions requires further investigation and more detailed studies. In particular, the development of new population genomics approaches is required in testing the existence of the extinct species, which served as the source of mtA‐type mitochondrial genomes.

## Conclusion

In this article, we put forward the argument that sfC and sfR should be regarded as host‐plant strains, rather than allochronic strains or genetic strains, as supported by recent population genomics studies that demonstrate a pattern of genomic differentiation according to host‐plants. We acknowledge that there are still many aspects of divergent evolution that remain underexplored, such as the relative contribution of the intrinsic reproductive barrier (Pashley & Martin, 1987; Velásquez‐Vélez *et al.*, 2011; Dumas *et al.*, 2015), different sexual pheromone blends (Groot *et al.*, 2008; Unbehend *et al.*, 2013, 2014), allochronic mating times (Pashley *et al.*, 1992; Schöfl *et al.*, 2011, 2009; Hänniger *et al.*, 2017; Tessnow *et al.*, 2022), and mitochondrial evolution to incipient evolution, as well as the interaction with host‐plant adaptation. Careful genomic studies seem to be indispensable to have a comprehensive view of the evolutionary history of incipient speciation in the FAW through the inference of the interaction between host‐plant adaptation and these divergent evolutionary forces. Nevertheless, host‐plant adaptation appears to be a primary driving force that plays a prominent role in incipient speciation in FAW, as widely observed in phytophagous insects (Nosil *et al.*, 2002).

## Disclosure

The authors declare that there is no conflict of interest.
